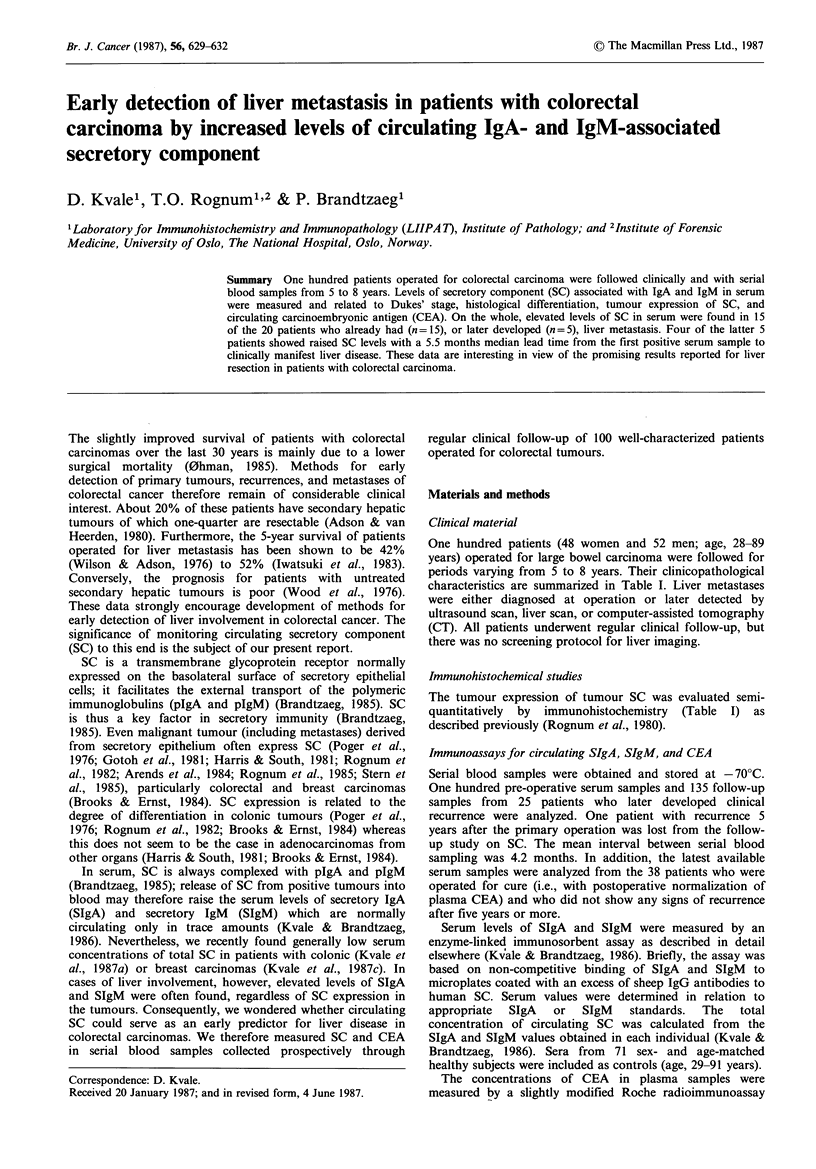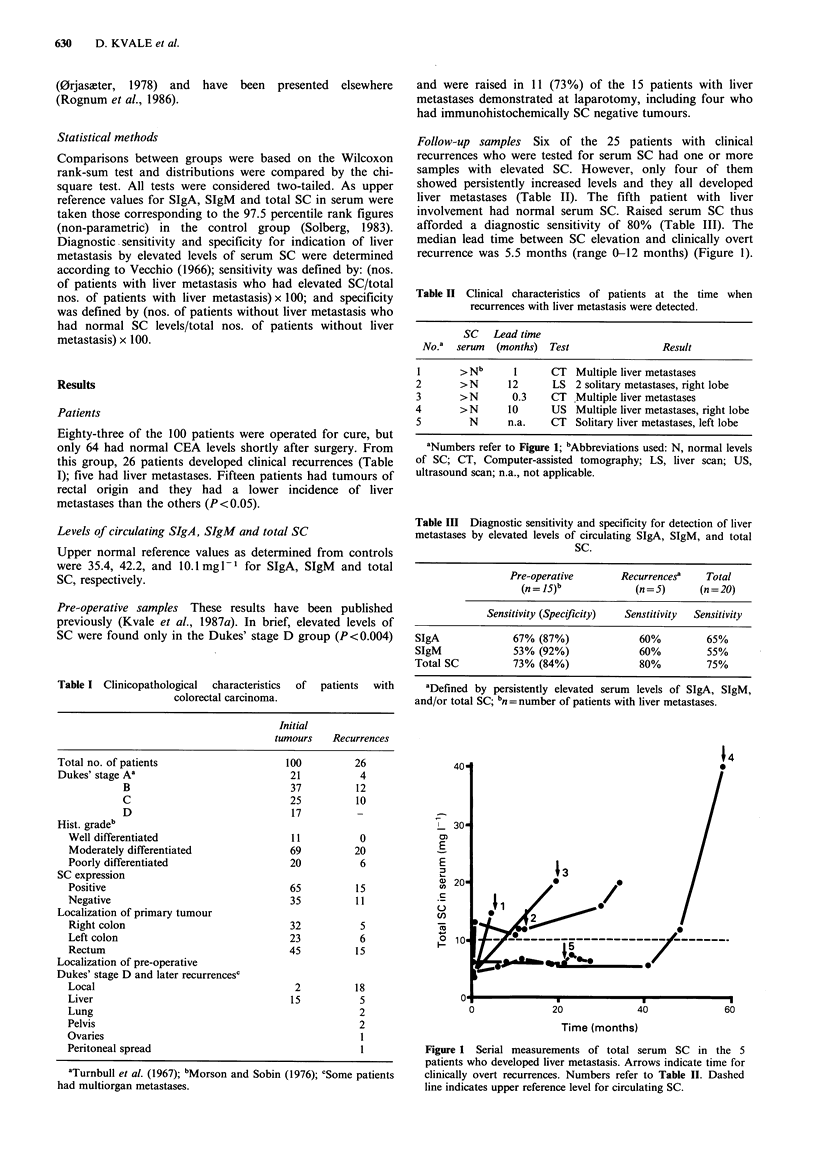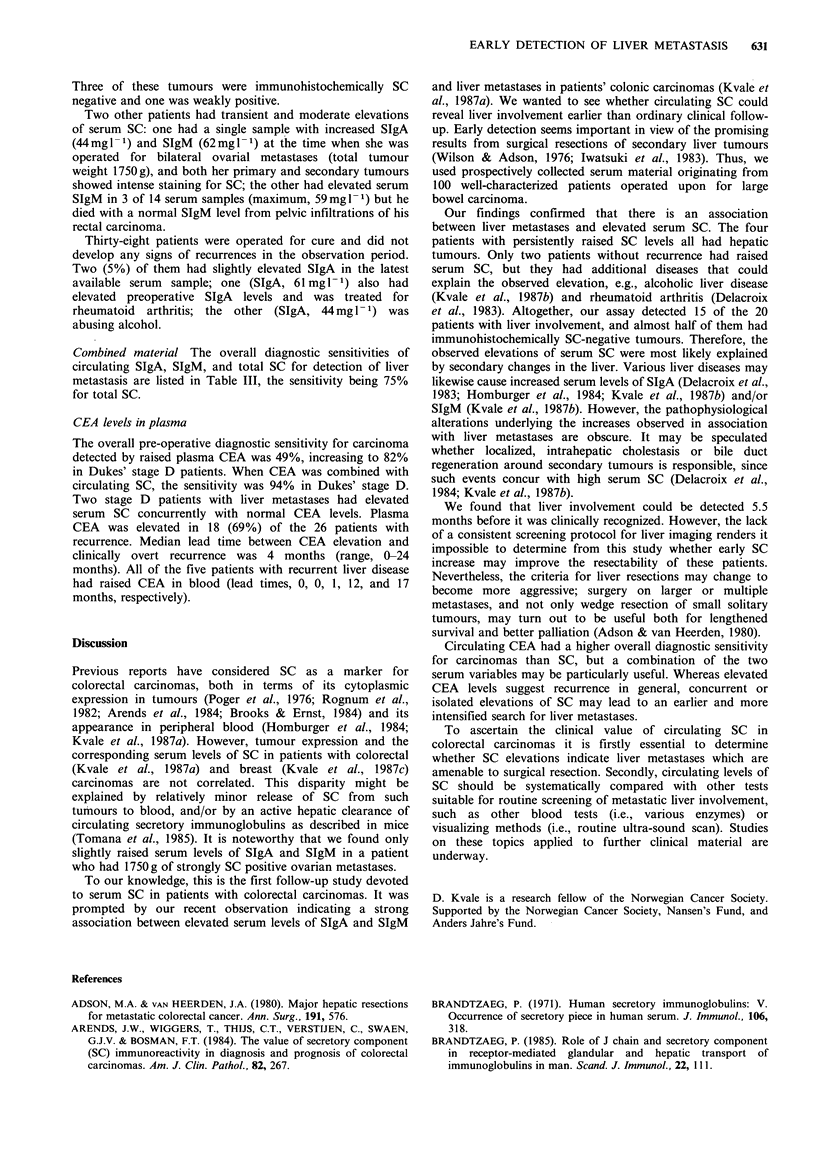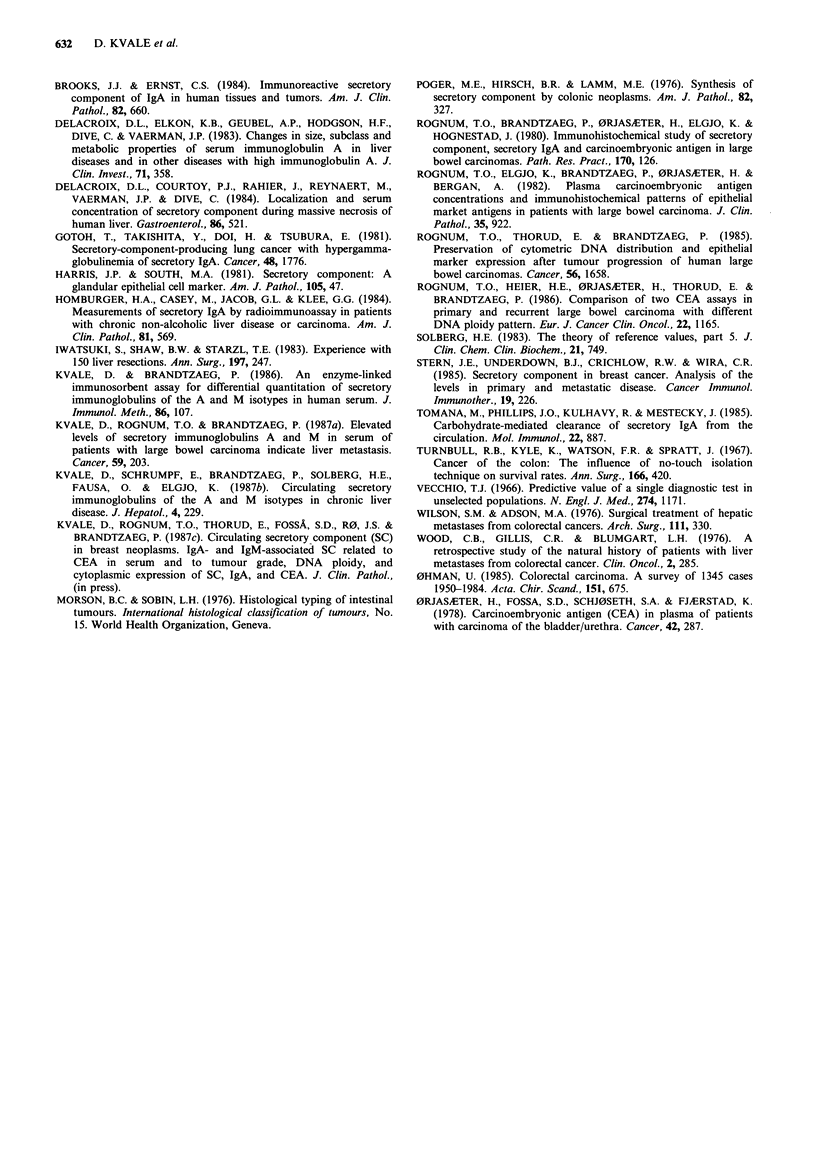# Early detection of liver metastasis in patients with colorectal carcinoma by increased levels of circulating IgA- and IgM-associated secretory component.

**DOI:** 10.1038/bjc.1987.256

**Published:** 1987-11

**Authors:** D. Kvale, T. O. Rognum, P. Brandtzaeg

**Affiliations:** Institute of Pathology, University of Oslo, National Hospital, Norway.

## Abstract

One hundred patients operated for colorectal carcinoma were followed clinically and with serial blood samples from 5 to 8 years. Levels of secretory component (SC) associated with IgA and IgM in serum were measured and related to Dukes' stage, histological differentiation, tumour expression of SC, and circulating carcinoembryonic antigen (CEA). On the whole, elevated levels of SC in serum were found in 15 of the 20 patients who already had (n = 15), or later developed (n = 5), liver metastasis. Four of the latter 5 patients showed raised SC levels with a 5.5 months median lead time from the first positive serum sample to clinically manifest liver disease. These data are interesting in view of the promising results reported for liver resection in patients with colorectal carcinoma.


Br. J. Cancer (1987), 56, 629–632 © The Macmillan Press Ltd., 1987

### Early detection of liver metastasis in patients with colorectal carcinoma by increased levels of circulating IgA- and IgM-associated secretory component

D. Kvale[1], T.O. Rognum[1,2] & P. Brandtzaeg[1]

[1]*Laboratory for Immunohistochemistry and Immunopathology (LIIPAT), Institute of Pathology; and* [2]*Institute of Forensic Medicine, University of Oslo, The National Hospital, Oslo, Norway.*

**Summary** One hundred patients operated for colorectal carcinoma were followed clinically and with serial blood samples from 5 to 8 years. Levels of secretory component (SC) associated with IgA and IgM in serum were measured and related to Dukes' stage, histological differentiation, tumour expression of SC, and circulating carcinoembryonic antigen (CEA). On the whole, elevated levels of SC in serum were found in 15 of the 20 patients who already had ($n=15$), or later developed ($n=5$), liver metastasis. Four of the latter 5 patients showed raised SC levels with a 5.5 months median lead time from the first positive serum sample to clinically manifest liver disease. These data are interesting in view of the promising results reported for liver resection in patients with colorectal carcinoma.

The slightly improved survival of patients with colorectal carcinomas over the last 30 years is mainly due to a lower surgical mortality (Øhman, 1985). Methods for early detection of primary tumours, recurrences, and metastases of colorectal cancer therefore remain of considerable clinical interest. About 20% of these patients have secondary hepatic tumours of which one-quarter are resectable (Adson & van Heerden, 1980). Furthermore, the 5-year survival of patients operated for liver metastasis has been shown to be 42% (Wilson & Adson, 1976) to 52% (Iwatsuki *et al.*, 1983). Conversely, the prognosis for patients with untreated secondary hepatic tumours is poor (Wood *et al.*, 1976). These data strongly encourage development of methods for early detection of liver involvement in colorectal cancer. The significance of monitoring circulating secretory component (SC) to this end is the subject of our present report.

SC is a transmembrane glycoprotein receptor normally expressed on the basolateral surface of secretory epithelial cells; it facilitates the external transport of the polymeric immunoglobulins (pIgA and pIgM) (Brandtzaeg, 1985). SC is thus a key factor in secretory immunity (Brandtzaeg, 1985). Even malignant tumour (including metastases) derived from secretory epithelium often express SC (Poger *et al.*, 1976; Gotoh *et al.*, 1981; Harris & South, 1981; Rognum *et al.*, 1982; Arends *et al.*, 1984; Rognum *et al.*, 1985; Stern *et al.*, 1985), particularly colorectal and breast carcinomas (Brooks & Ernst, 1984). SC expression is related to the degree of differentiation in colonic tumours (Poger *et al.*, 1976; Rognum *et al.*, 1982; Brooks & Ernst, 1984) whereas this does not seem to be the case in adenocarcinomas from other organs (Harris & South, 1981; Brooks & Ernst, 1984).

In serum, SC is always complexed with pIgA and pIgM (Brandtzaeg, 1985); release of SC from positive tumours into blood may therefore raise the serum levels of secretory IgA (SIgA) and secretory IgM (SIgM) which are normally circulating only in trace amounts (Kvale & Brandtzaeg, 1986). Nevertheless, we recently found generally low serum concentrations of total SC in patients with colonic (Kvale *et al.*, 1987*a*) or breast carcinomas (Kvale *et al.*, 1987*c*). In cases of liver involvement, however, elevated levels of SIgA and SIgM were often found, regardless of SC expression in the tumours. Consequently, we wondered whether circulating SC could serve as an early predictor for liver disease in colorectal carcinomas. We therefore measured SC and CEA in serial blood samples collected prospectively through regular clinical follow-up of 100 well-characterized patients operated for colorectal tumours.

Correspondence: D. Kvale.
Received 20 January 1987; and in revised form, 4 June 1987.

### Materials and methods

### Clinical material

One hundred patients (48 women and 52 men; age, 28–89 years) operated for large bowel carcinoma were followed for periods varying from 5 to 8 years. Their clinicopathological characteristics are summarized in Table I. Liver metastases were either diagnosed at operation or later detected by ultrasound scan, liver scan, or computer-assisted tomography (CT). All patients underwent regular clinical follow-up, but there was no screening protocol for liver imaging.

### Immunohistochemical studies

The tumour expression of tumour SC was evaluated semi-quantitatively by immunohistochemistry (Table I) as described previously (Rognum *et al.*, 1980).

### Immunoassays for circulating SIgA, SIgM, and CEA

Serial blood samples were obtained and stored at −70°C. One hundred pre-operative serum samples and 135 follow-up samples from 25 patients who later developed clinical recurrence were analyzed. One patient with recurrence 5 years after the primary operation was lost from the follow-up study on SC. The mean interval between serial blood sampling was 4.2 months. In addition, the latest available serum samples were analyzed from the 38 patients who were operated for cure (i.e., with postoperative normalization of plasma CEA) and who did not show any signs of recurrence after five years or more.

Serum levels of SIgA and SIgM were measured by an enzyme-linked immunosorbent assay as described in detail elsewhere (Kvale & Brandtzaeg, 1986). Briefly, the assay was based on non-competitive binding of SIgA and SIgM to microplates coated with an excess of sheep IgG antibodies to human SC. Serum values were determined in relation to appropriate SIgA or SIgM standards. The total concentration of circulating SC was calculated from the SIgA and SIgM values obtained in each individual (Kvale & Brandtzaeg, 1986). Sera from 71 sex- and age-matched healthy subjects were included as controls (age, 29–91 years).

The concentrations of CEA in plasma samples were measured by a slightly modified Roche radioimmunoassay

(Ørjasæter, 1978) and have been presented elsewhere (Rognum et al., 1986).

*Statistical methods*

Comparisons between groups were based on the Wilcoxon rank-sum test and distributions were compared by the chi-square test. All tests were considered two-tailed. As upper reference values for SIgA, SIgM and total SC in serum were taken those corresponding to the 97.5 percentile rank figures (non-parametric) in the control group (Solberg, 1983). Diagnostic sensitivity and specificity for indication of liver metastasis by elevated levels of serum SC were determined according to Vecchio (1966); sensitivity was defined by: (nos. of patients with liver metastasis who had elevated SC/total nos. of patients with liver metastasis) × 100; and specificity was defined by (nos. of patients without liver metastasis who had normal SC levels/total nos. of patients without liver metastasis) × 100.

### Results

*Patients*

Eighty-three of the 100 patients were operated for cure, but only 64 had normal CEA levels shortly after surgery. From this group, 26 patients developed clinical recurrences (Table I); five had liver metastases. Fifteen patients had tumours of rectal origin and they had a lower incidence of liver metastases than the others ($P<0.05$).

*Levels of circulating SIgA, SIgM and total SC*

Upper normal reference values as determined from controls were 35.4, 42.2, and 10.1 $mg\,l^{-1}$ for SIgA, SIgM and total SC, respectively.

*Pre-operative samples* These results have been published previously (Kvale et al., 1987a). In brief, elevated levels of SC were found only in the Dukes' stage D group ($P<0.004$) and were raised in 11 (73%) of the 15 patients with liver metastases demonstrated at laparotomy, including four who had immunohistochemically SC negative tumours.

**Table I** Clinicopathological characteristics of patients with colorectal carcinoma.

| | Initial tumours | Recurrences |
|---|---|---|
| Total no. of patients | 100 | 26 |
| Dukes' stage A[a] | 21 | 4 |
| B | 37 | 12 |
| C | 25 | 10 |
| D | 17 | - |
| Hist. grade[b] | | |
| Well differentiated | 11 | 0 |
| Moderately differentiated | 69 | 20 |
| Poorly differentiated | 20 | 6 |
| SC expression | | |
| Positive | 65 | 15 |
| Negative | 35 | 11 |
| Localization of primary tumour | | |
| Right colon | 32 | 5 |
| Left colon | 23 | 6 |
| Rectum | 45 | 15 |
| Localization of pre-operative Dukes' stage D and later recurrences[c] | | |
| Local | 2 | 18 |
| Liver | 15 | 5 |
| Lung | | 2 |
| Pelvis | | 2 |
| Ovaries | | 1 |
| Peritoneal spread | | 1 |

[a]Turnbull et al. (1967); [b]Morson and Sobin (1976); [c]Some patients had multiorgan metastases.

*Follow-up samples* Six of the 25 patients with clinical recurrences who were tested for serum SC had one or more samples with elevated SC. However, only four of them showed persistently increased levels and they all developed liver metastases (Table II). The fifth patient with liver involvement had normal serum SC. Raised serum SC thus afforded a diagnostic sensitivity of 80% (Table III). The median lead time between SC elevation and clinically overt recurrence was 5.5 months (range 0–12 months) (Figure 1).

**Table II** Clinical characteristics of patients at the time when recurrences with liver metastasis were detected.

| No.[a] | SC serum | Lead time (months) | Test | Result |
|---|---|---|---|---|
| 1 | >N[b] | 1 | CT | Multiple liver metastases |
| 2 | >N | 12 | LS | 2 solitary metastases, right lobe |
| 3 | >N | 0.3 | CT | Multiple liver metastases |
| 4 | >N | 10 | US | Multiple liver metastases, right lobe |
| 5 | N | n.a. | CT | Solitary liver metastases, left lobe |

[a]Numbers refer to **Figure 1**; [b]Abbreviations used: N, normal levels of SC; CT, Computer-assisted tomography; LS, liver scan; US, ultrasound scan; n.a., not applicable.

**Table III** Diagnostic sensitivity and specificity for detection of liver metastases by elevated levels of circulating SIgA, SIgM, and total SC.

| | Pre-operative ($n=15$)[b] | Recurrences[a] ($n=5$) | Total ($n=20$) |
|---|---|---|---|
| | Sensitivity (Specificity) | Senstitivity | Sensitivity |
| SIgA | 67% (87%) | 60% | 65% |
| SIgM | 53% (92%) | 60% | 55% |
| Total SC | 73% (84%) | 80% | 75% |

[a]Defined by persistently elevated serum levels of SIgA, SIgM, and/or total SC; [b]$n$ = number of patients with liver metastases.

**Figure 1** Serial measurements of total serum SC in the 5 patients who developed liver metastasis. Arrows indicate time for clinically overt recurrences. Numbers refer to **Table II**. Dashed line indicates upper reference level for circulating SC.

Three of these tumours were immunohistochemically SC negative and one was weakly positive.

Two other patients had transient and moderate elevations of serum SC: one had a single sample with increased SIgA ($44 mg l^{-1}$) and SIgM ($62 mg l^{-1}$) at the time when she was operated for bilateral ovarial metastases (total tumour weight 1750 g), and both her primary and secondary tumours showed intense staining for SC; the other had elevated serum SIgM in 3 of 14 serum samples (maximum, $59 mg l^{-1}$) but he died with a normal SIgM level from pelvic infiltrations of his rectal carcinoma.

Thirty-eight patients were operated for cure and did not develop any signs of recurrences in the observation period. Two (5%) of them had slightly elevated SIgA in the latest available serum sample; one (SIgA, $61 mg l^{-1}$) also had elevated preoperative SIgA levels and was treated for rheumatoid arthritis; the other (SIgA, $44 mg l^{-1}$) was abusing alcohol.

*Combined material* The overall diagnostic sensitivities of circulating SIgA, SIgM, and total SC for detection of liver metastasis are listed in Table III, the sensitivity being 75% for total SC.

*CEA levels in plasma*

The overall pre-operative diagnostic sensitivity for carcinoma detected by raised plasma CEA was 49%, increasing to 82% in Dukes' stage D patients. When CEA was combined with circulating SC, the sensitivity was 94% in Dukes' stage D. Two stage D patients with liver metastases had elevated serum SC concurrently with normal CEA levels. Plasma CEA was elevated in 18 (69%) of the 26 patients with recurrence. Median lead time between CEA elevation and clinically overt recurrence was 4 months (range, 0–24 months). All of the five patients with recurrent liver disease had raised CEA in blood (lead times, 0, 0, 1, 12, and 17 months, respectively).

### Discussion

Previous reports have considered SC as a marker for colorectal carcinomas, both in terms of its cytoplasmic expression in tumours (Poger *et al.*, 1976; Rognum *et al.*, 1982; Arends *et al.*, 1984; Brooks & Ernst, 1984) and its appearance in peripheral blood (Homburger *et al.*, 1984; Kvale *et al.*, 1987*a*). However, tumour expression and the corresponding serum levels of SC in patients with colorectal (Kvale *et al.*, 1987*a*) and breast (Kvale *et al.*, 1987*c*) carcinomas are not correlated. This disparity might be explained by relatively minor release of SC from such tumours to blood, and/or by an active hepatic clearance of circulating secretory immunoglobulins as described in mice (Tomana *et al.*, 1985). It is noteworthy that we found only slightly raised serum levels of SIgA and SIgM in a patient who had 1750 g of strongly SC positive ovarian metastases.

To our knowledge, this is the first follow-up study devoted to serum SC in patients with colorectal carcinomas. It was prompted by our recent observation indicating a strong association between elevated serum levels of SIgA and SIgM and liver metastases in patients' colonic carcinomas (Kvale *et al.*, 1987*a*). We wanted to see whether circulating SC could reveal liver involvement earlier than ordinary clinical follow-up. Early detection seems important in view of the promising results from surgical resections of secondary liver tumours (Wilson & Adson, 1976; Iwatsuki *et al.*, 1983). Thus, we used prospectively collected serum material originating from 100 well-characterized patients operated upon for large bowel carcinoma.

Our findings confirmed that there is an association between liver metastases and elevated serum SC. The four patients with persistently raised SC levels all had hepatic tumours. Only two patients without recurrence had raised serum SC, but they had additional diseases that could explain the observed elevation, e.g., alcoholic liver disease (Kvale *et al.*, 1987*b*) and rheumatoid arthritis (Delacroix *et al.*, 1983). Altogether, our assay detected 15 of the 20 patients with liver involvement, and almost half of them had immunohistochemically SC-negative tumours. Therefore, the observed elevations of serum SC were most likely explained by secondary changes in the liver. Various liver diseases may likewise cause increased serum levels of SIgA (Delacroix *et al.*, 1983; Homburger *et al.*, 1984; Kvale *et al.*, 1987*b*) and/or SIgM (Kvale *et al.*, 1987*b*). However, the pathophysiological alterations underlying the increases observed in association with liver metastases are obscure. It may be speculated whether localized, intrahepatic cholestasis or bile duct regeneration around secondary tumours is responsible, since such events concur with high serum SC (Delacroix *et al.*, 1984; Kvale *et al.*, 1987*b*).

We found that liver involvement could be detected 5.5 months before it was clinically recognized. However, the lack of a consistent screening protocol for liver imaging renders it impossible to determine from this study whether early SC increase may improve the resectability of these patients. Nevertheless, the criteria for liver resections may change to become more aggressive; surgery on larger or multiple metastases, and not only wedge resection of small solitary tumours, may turn out to be useful both for lengthened survival and better palliation (Adson & van Heerden, 1980).

Circulating CEA had a higher overall diagnostic sensitivity for carcinomas than SC, but a combination of the two serum variables may be particularly useful. Whereas elevated CEA levels suggest recurrence in general, concurrent or isolated elevations of SC may lead to an earlier and more intensified search for liver metastases.

To ascertain the clinical value of circulating SC in colorectal carcinomas it is firstly essential to determine whether SC elevations indicate liver metastases which are amenable to surgical resection. Secondly, circulating levels of SC should be systematically compared with other tests suitable for routine screening of metastatic liver involvement, such as other blood tests (i.e., various enzymes) or visualizing methods (i.e., routine ultra-sound scan). Studies on these topics applied to further clinical material are underway.

D. Kvale is a research fellow of the Norwegian Cancer Society. Supported by the Norwegian Cancer Society, Nansen's Fund, and Anders Jahre's Fund.

### References

ADSON, M.A. & VAN HEERDEN, J.A. (1980). Major hepatic resections for metastatic colorectal cancer. *Ann. Surg.*, **191**, 576.

ARENDS, J.W., WIGGERS, T., THIJS, C.T., VERSTIJEN, C., SWAEN, G.J.V. & BOSMAN, F.T. (1984). The value of secretory component (SC) immunoreactivity in diagnosis and prognosis of colorectal carcinomas. *Am. J. Clin. Pathol.*, **82**, 267.

BRANDTZAEG, P. (1971). Human secretory immunoglobulins: V. Occurrence of secretory piece in human serum. *J. Immunol.*, **106**, 318.

BRANDTZAEG, P. (1985). Role of J chain and secretory component in receptor-mediated glandular and hepatic transport of immunoglobulins in man. *Scand. J. Immunol.*, **22**, 111.

BROOKS, J.J. & ERNST, C.S. (1984). Immunoreactive secretory component of IgA in human tissues and tumors. *Am. J. Clin. Pathol.*, **82,** 660.

DELACROIX, D.L., ELKON, K.B., GEUBEL, A.P., HODGSON, H.F., DIVE, C. & VAERMAN, J.P. (1983). Changes in size, subclass and metabolic properties of serum immunoglobulin A in liver diseases and in other diseases with high immunoglobulin A. *J. Clin. Invest.*, **71,** 358.

DELACROIX, D.L., COURTOY, P.J., RAHIER, J., REYNAERT, M., VAERMAN, J.P. & DIVE, C. (1984). Localization and serum concentration of secretory component during massive necrosis of human liver. *Gastroenterol.*, **86,** 521.

GOTOH, T., TAKISHITA, Y., DOI, H. & TSUBURA, E. (1981). Secretory-component-producing lung cancer with hypergammaglobulinemia of secretory IgA. *Cancer*, **48,** 1776.

HARRIS, J.P. & SOUTH, M.A. (1981). Secretory component: A glandular epithelial cell marker. *Am. J. Pathol.*, **105,** 47.

HOMBURGER, H.A., CASEY, M., JACOB, G.L. & KLEE, G.G. (1984). Measurements of secretory IgA by radioimmunoassay in patients with chronic non-alcoholic liver disease or carcinoma. *Am. J. Clin. Pathol.*, **81,** 569.

IWATSUKI, S., SHAW, B.W. & STARZL, T.E. (1983). Experience with 150 liver resections. *Ann. Surg.*, **197,** 247.

KVALE, D. & BRANDTZAEG, P. (1986). An enzyme-linked immunosorbent assay for differential quantitation of secretory immunoglobulins of the A and M isotypes in human serum. *J. Immunol. Meth.*, **86,** 107.

KVALE, D., ROGNUM, T.O. & BRANDTZAEG, P. (1987*a*). Elevated levels of secretory immunoglobulins A and M in serum of patients with large bowel carcinoma indicate liver metastasis. *Cancer*, **59,** 203.

KVALE, D., SCHRUMPF, E., BRANDTZAEG, P., SOLBERG, H.E., FAUSA, O. & ELGJO, K. (1987*b*). Circulating secretory immunoglobulins of the A and M isotypes in chronic liver disease. *J. Hepatol.*, **4,** 229.

KVALE, D., ROGNUM, T.O., THORUD, E., FOSSÅ, S.D., RØ, J.S. & BRANDTZAEG, P. (1987*c*). Circulating secretory component (SC) in breast neoplasms. IgA- and IgM-associated SC related to CEA in serum and to tumour grade, DNA ploidy, and cytoplasmic expression of SC, IgA, and CEA. *J. Clin. Pathol.*, (in press).

MORSON, B.C. & SOBIN, L.H. (1976). Histological typing of intestinal tumours. *International histological classification of tumours*, No. 15. World Health Organization, Geneva.

POGER, M.E., HIRSCH, B.R. & LAMM, M.E. (1976). Synthesis of secretory component by colonic neoplasms. *Am. J. Pathol.*, **82,** 327.

ROGNUM, T.O., BRANDTZAEG, P., ØRJASÆTER, H., ELGJO, K. & HOGNESTAD, J. (1980). Immunohistochemical study of secretory component, secretory IgA and carcinoembryonic antigen in large bowel carcinomas. *Path. Res. Pract.*, **170,** 126.

ROGNUM, T.O., ELGJO, K., BRANDTZAEG, P., ØRJASÆTER, H. & BERGAN, A. (1982). Plasma carcinoembryonic antigen concentrations and immunohistochemical patterns of epithelial market antigens in patients with large bowel carcinoma. *J. Clin. Pathol.*, **35,** 922.

ROGNUM, T.O., THORUD, E. & BRANDTZAEG, P. (1985). Preservation of cytometric DNA distribution and epithelial marker expression after tumour progression of human large bowel carcinomas. *Cancer*, **56,** 1658.

ROGNUM, T.O., HEIER, H.E., ØRJASÆTER, H., THORUD, E. & BRANDTZAEG, P. (1986). Comparison of two CEA assays in primary and recurrent large bowel carcinoma with different DNA ploidy pattern. *Eur. J. Cancer Clin. Oncol.*, **22,** 1165.

SOLBERG, H.E. (1983). The theory of reference values, part 5. *J. Clin. Chem. Clin. Biochem.*, **21,** 749.

STERN, J.E., UNDERDOWN, B.J., CRICHLOW, R.W. & WIRA, C.R. (1985). Secretory component in breast cancer. Analysis of the levels in primary and metastatic disease. *Cancer Immunol. Immunother.*, **19,** 226.

TOMANA, M., PHILLIPS, J.O., KULHAVY, R. & MESTECKY, J. (1985). Carbohydrate-mediated clearance of secretory IgA from the circulation. *Mol. Immunol.*, **22,** 887.

TURNBULL, R.B., KYLE, K., WATSON, F.R. & SPRATT, J. (1967). Cancer of the colon: The influence of no-touch isolation technique on survival rates. *Ann. Surg.*, **166,** 420.

VECCHIO, T.J. (1966). Predictive value of a single diagnostic test in unselected populations. *N. Engl. J. Med.*, **274,** 1171.

WILSON, S.M. & ADSON, M.A. (1976). Surgical treatment of hepatic metastases from colorectal cancers. *Arch. Surg.*, **111,** 330.

WOOD, C.B., GILLIS, C.R. & BLUMGART, L.H. (1976). A retrospective study of the natural history of patients with liver metastases from colorectal cancer. *Clin. Oncol.*, **2,** 285.

ØHMAN, U. (1985). Colorectal carcinoma. A survey of 1345 cases 1950–1984. *Acta. Chir. Scand.*, **151,** 675.

ØRJASÆTER, H., FOSSA, S.D., SCHJØSETH, S.A. & FJÆRSTAD, K. (1978). Carcinoembryonic antigen (CEA) in plasma of patients with carcinoma of the bladder/urethra. *Cancer*, **42,** 287.